# How influencers motivate inactive adolescents to be more physically active: a mixed methods study

**DOI:** 10.3389/fpubh.2024.1429850

**Published:** 2024-09-17

**Authors:** Rahel Aschwanden, Claude Messner

**Affiliations:** Department of Consumer Behavior, Institute of Marketing and Management, University of Bern, Bern, Switzerland

**Keywords:** physical activity, adolescents, influencers, social media, health promotion, health behavior

## Abstract

**Introduction:**

Regular physical activity offers numerous health benefits, particularly for adolescents. However, only 14% of school-aged children in Switzerland achieve the World Health Organization's recommendation of 60 min of moderate to vigorous physical activity per day. Changing health behaviors is a complex process in which understanding behavioral and communication patterns is crucial. Because adolescents spend substantial time on social media channels and obtain information from them, these are potential channels for accessing health-related content. This study explores the questions of which influencers and what content motivate adolescents to be more physically active and whether influencers can impact enjoyment and the intention to engage in physical activity.

**Methods:**

The study employed a convergent mixed methods approach, combining self-assessment questionnaires and semi-structured interviews. Ninety-three adolescents aged 14–20 years who exercised < 1 h per day participated. They followed one of the six participating influencers on Instagram. Over 6 weeks, the questionnaires collected quantitative data, measuring enjoyment, stages of change, and physical activity levels. Furthermore, semi-structured interviews were conducted with 23 adolescents and six influencers to gain in-depth insights.

**Results:**

The quantitative findings indicate that adolescent followers enjoyed physical activity more after the social media intervention and at follow-up than at the beginning of the study. The followers' stages of change progressed over time. Compared with those following more athletic influencers, followers of nonathletic influencers (that is, a singer, a journalist, and an eFootballer) showed increased physical activity over time. Qualitative analysis highlighted Instagram factors influencing physical activity, including resonance with influencers' lifestyles and preference for simple, relatable activities. Authentic content was positively associated with increased exercise, particularly among already motivated followers.

**Discussion:**

Unexpectedly, nonathletic influencers, such as a singer, a journalist, and an eFootballer, motivated adolescents best despite their nontraditional focus on physical activity. Their success stems from relatable lifestyles and simple activities that are easily incorporated into their daily routines. Conversely, athletic influencers demonstrated challenging exercises that were fascinating but difficult to adopt. This finding suggests the potential for utilizing nonathletic influencers in future campaigns targeting inactive adolescents.

## 1 Introduction

Regular physical activity in adolescents is associated with improved physical, cognitive, social and overall mental health (1–3). Globally, however, 81% of adolescents aged 11–17 years do not meet the recommended minimum of 60 min of moderate to vigorous physical activity per day (4). Instead, adolescents are increasingly using social media platforms (5), acquiring health-related information from them (6) that influences their behavior and decision-making (7). In Switzerland, adolescents use their mobile phones for an average of 3 h and 33 min on weekdays and 4 h and 53 min per day on weekends (8), with 86% of them using social networks multiple times a day (9), with Instagram being the most popular platform (10).

The present study aimed to investigate the potential of influencers in promoting physical activity among physically inactive adolescents in Switzerland.

The utilization of influencers in brand and product promotion as well as health communication is a well-established practice (11–17, 86). Influencers, also referred to as “opinion leaders”, have a significant impact on their followers, influencing their attitudes, behaviors, and decisions (13, 18–21). In influencer marketing, most of the promoted products do not foster followers' health and wellbeing, but rather unhealthy products, that is, food and beverages high in fat, sugar, and/or salt (5, 22). In contrast, in health communication, influencers can, for example in the fitness scene, positively influence the behavior of sports enthusiasts (23–25). Because followers prefer to follow influencers who share the same preferences and lifestyle (26), fitness influencers tend to be followed by people who are interested in fitness and who already perform similar exercises. A crucial question remains as to whether inactive followers can also be motivated by influencers to increase their physical activity.

Influencers can play a key role in promoting physical activity among inactive adolescents for several reasons.

First, a major challenge in interventions targeting inactive individuals is the difficulty of reaching them (27). Considering that adolescents are the most active users of social networks (28), influencers emerge as a potential avenue to effectively engage and reach them with health communication (29). This is particularly important because, among sedentary adolescents, frequent use of social media is associated with a lower likelihood of engaging in vigorous daily physical activity (30).

Second, influencers have a deep understanding of their target audience, and they tailor their messages to align with the specific characteristics of that audience, which is important (31); they can adopt the perspective of their community and communicate with appropriate content. By openly sharing private aspects of their lives and engaging directly with their followers, they are perceived as more trustworthy and approachable than traditional advertising or celebrities (12, 32). Influencers create a sense of closeness with their followers by sharing authentic content in the areas in which they claim expertise (33). These interactions often lead to a one-way symbolic relationship between the influencer and the follower, a so-called parasocial relationship (34). Because of this proximity, influencers are considered to have motivational power. This motivational power of influencers is crucial because a lack of motivation is a major barrier for physically inactive adolescents (35).

The third rationale is based on social learning theory (36), which states that individuals learn by observing a model's behavior. Observational learning also applies to media consumption (37). As with traditional media figures, influencers serve as models and can, therefore, guide or change the audience's behavior, drawing a parallel between historical media figures and modern influencers in terms of their role in social learning. The original media effect model perceives audiences as passive recipients of media, merely absorbing content (38). Social media has transformed the passive role of recipients into that of active contributors (5). Everyone can express their opinions and have the power to influence others (39). Several studies have shown that social media can influence behavior, opinions, or attitudes positively or negatively (7, 25, 40–42). Observing a behavior can also prompt people to engage in physical activity, especially inactive people who recognize the positive nature of physical activity but lack the ultimate motivation to engage (25). A media figure can encourage a particular behavior by demonstrating its positive outcomes (43) and emotions (23), such as stress reduction or enjoyment in a group setting. This phenomenon is used in product advertising when desirable outcomes, such as beauty or success, are shown (25).

**Figure 1:**
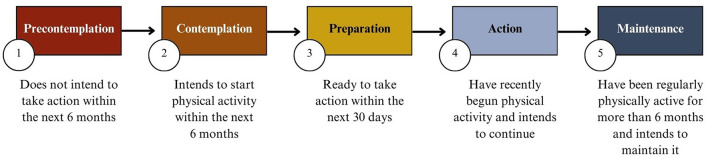
The stages of behavior change according to the transtheoretical model (44) (illustration by the authors).

Physically inactive adolescents differ in their following behaviors on social media, with some following nonsporting influencers such as singers and others also following sporting influencers who provide entertaining content. The intention to change their inactivity also varies, as indicated by the stages of behavior change in the transtheoretical model (44) (Figure 1). The transtheoretical model shows that individuals progress through the five stages of behavior change, and that they may relapse to a previous stage. In the precontemplation stage, individuals do not plan to engage in regular physical activity in the next 6 months. In the contemplation stage, individuals intend to start regular physical activity within the next 6 months. Those in the preparation stage are ready to start regular physical activity within the next 30 days. Individuals in the action stage have already incorporated regular physical activity into their lives in the last 6 months. Individuals who are in the maintenance stage have been engaging in regular physical activity for a longer period, usually for more than 6 months. According to the five stages, physically inactive individuals are in the precontemplation, contemplation, or preparation stages.

The present study examines the potential of influencers to motivate inactive adolescents to increase their physical activity. To achieve this goal, we gathered and analyzed physical activity data and conducted semi-structured in-depth interviews to obtain a deeper understanding of the communication behaviors of influencers and adolescents in the context of physical activity. The dual focus of examining influencers and followers' behaviors allows for a comprehensive understanding of the dynamics at play. Previous research on promoting effective health communication on social media highlights the potential for influencers to adapt their strategies to effectively communicate health-related messages (45).

Using the transtheoretical model of behavior change (44), our focus is on the physically inactive target group; that is, on adolescents in the precontemplation, contemplation, and preparation stages. Over 1 month, six influencers aimed to motivate adolescents aged 14–20 years to increase their physical activity levels. Regardless of their interest in sports, the influencers shared diverse content that promoted physical activity. The present study addresses the following three research questions:

RQ1_quan+qual_: Can influencers enhance the enjoyment of physical activity in adolescents?
RQ2_quan+qual_: Can influencers change the intention to engage in physical activity in adolescents?
RQ3_quan+qual_: Which influencer characteristics and content inspire adolescents to be more physically active?

The question of whether influencers can effectively motivate sedentary adolescents to become more active remains open. Encouraging inactive followers to increase their physical activity is significantly more challenging than promoting product purchases. Buying a product is a one-time behavior, often triggered by a spontaneous click on a sale button that provides immediate gratification. In contrast, increasing physical activity involves developing a habit over a prolonged period. Typically, the nonathletic target group does not initially find physical activity enjoyable (46); positive feelings associated with exercise need time to develop. It is uncertain whether influencers can foster an enjoyment of exercise among their followers or influence their intentions to engage in physical activity more frequently. Furthermore, even if influencers can inspire a short-term increase in physical activity, it is unclear whether this effect can be sustained in the long term. Given that influencers are a heterogeneous group, it is essential to identify which characteristics are most effective in changing followers' exercise habits. For instance, an influencer's athleticism or authenticity might play a crucial role in motivating followers to be more active.

## 2 Methods

The present study employed a QUAN + QUAL convergent mixed methods design, where the results were analyzed separately and then integrated (47–49). The integration of quantitative and qualitative approaches creates a more holistic understanding (50). To test whether enjoyment of physical activity, the stages of change, and physical activity increased during the intervention period, we collected and analyzed the questionnaire data. To develop an in-depth understanding of how followers were impacted by influencers, we conducted semi-structured in-depth interviews with followers and influencers.

### 2.1 Sample

#### 2.1.1 Influencers

Six influencers of diverse genders, ages, and backgrounds were recruited via the project team's network (33% female, 67% male; *M*_*Age*_ = 24.8, *SD*_*Age*_ = 4.5; see Table 1). Important criteria for their selection were that the influencers identified with the health-promoting theme, were motivated to support adolescents, and came from diverse domains. They should be regarded as trusted tastemakers in sports or another field (12). The number of followers was less important, with influencers having between 5.6k and 70.7k followers (see Table 1). All the influencers were Swiss and lived in German-speaking Switzerland. Each influencer was compensated with 6,000 CHF (~$6,653) for their contributions.

**Table 1:** Characteristics of the six influencers (Status: August 2022).

**Influencers**	**Gender**	**Age**	**Background**	**Number of followers**
Influencer 1	Female	27	Nonathlete: Singer and songwriter	10.2k
Influencer 2	Female	28	Nonathlete: Journalist, event organizer	9.3k
Influencer 3	Male	27	Nonathlete: eFootballer	5.6k
Influencer 4	Male	16	Athlete: Snowboarder	14.4k
Influencer 5	Male	27	Athlete: Snowboarder	70.7k
Influencer 6	Male	27	Athlete: Mountain biker, sports photographer	14k

The project team did not provide specific guidelines to the influencers regarding the number and content of posts on social media. The six influencers were free to interact authentically with their communities in unique ways, employing various strategies and communication tools.

Influencer 1 communicated with her followers several times a week using interactive stories. She authentically showed her life and often added a touch of humor with simple activities.

Influencer 2 described her daily routines, such as her morning yoga. She showed links to external sports videos that she also liked to imitate. She interacted with her community by asking, for example, what prevented her audience from exercising more.

Influencer 3, who is a professional gamer, often spent a significant amount of time sitting. He shared with his followers how he motivated himself. He showed them how he planned his week and kept fixed times free for sports, such as when he went to the gym.

Influencer 4 was a naturally sporty and talented young snowboarder who maintained almost daily activity on his social media platform to engage with his followers. He showcased impressive workout videos, such as performing flips on a trampoline, high-speed mountain biking through a forest, basketball and soccer skills, juggling, and playing gummi twist.

Influencer 5 primarily engaged with his community through group chats. He sought to provide motivational words by offering ideas, such as activities to do in hot weather. His goal was to help adolescents discover a physical activity that they enjoy and where they can find their passion, ideally by engaging in it together with a friend.

Influencer 6 shared bike challenge videos, starting with relatively simple videos and progressively increasing their complexity. The followers could send their bike videos to him for feedback. Toward the end of the project, he ran low on ideas, resulting in decreased engagement with his followers.

#### 2.1.2 Adolescent followers

Adolescents were recruited via Instagram. The six influencers and project team shared the University of Bern recruitment survey. The adolescents recruited by the project team were additionally asked about their areas of interest and assigned to follow one of the six influencers accordingly. Overall, 1,164 people clicked on the recruitment survey link. However, only 93 adolescent followers (69% female, 31% male, *M*_*Age*_ = 16.8, *SD*_*Age*_ = 1.7) met the following inclusion criteria: The participants were between 14 and 20 years of age, lived in Switzerland, and exercised moderately to vigorously for < 1 h per day. This means that we were interested in adolescents who were in the precontemplation, contemplation, or preparation stage and not already in the action or maintenance stage of the transtheoretical model (44). The age range of 14–20 years aligns with the definition of adolescence by the Federal Office of Sports in Switzerland. Traditionally, adolescents are defined as those between 10 and 19 years old (85). However, recent perspectives suggest extending this definition to encompass ages 10–24 years due to evolving understandings of biological maturation and delayed role transitions (51, 52). Despite recognizing the developmental differences between ages 14 and 20, our analysis did not reveal any evidence that the impact of influencers on younger adolescents differs from that of older adolescents. As our research does not focus on age-related research questions, we do not report any age effects.

As an incentive, followers received 95 CHF (~$105) for completing the daily questionnaire for 42 days. If they participated in an interview, they were rewarded with an additional 50 CHF (~$55).

The Ethics Committee of the Faculty of Business, Economics and Social Sciences of the University of Bern declared that the research project “Sportfluencer - Wie lnfluencer Jugendliche zu mehr Bewegung motivieren” complies with the ethical standards of the faculty. Ethical clearance was provided on May 2, 2022, with serial number 092022. The quantitative data was provided in the Supplementary material without identifying information. We obtained consent from all participants.

### 2.2 Procedure and design

The project started with a roundtable attended by the project team and the six specifically selected influencers. It was important to get to know each other and explain the goals and values of the project. The influencers were given input on behavior change theories the credible and reliable communication of health information. They had the opportunity to brainstorm together on content related to the project's goal.

**Figure 2:**
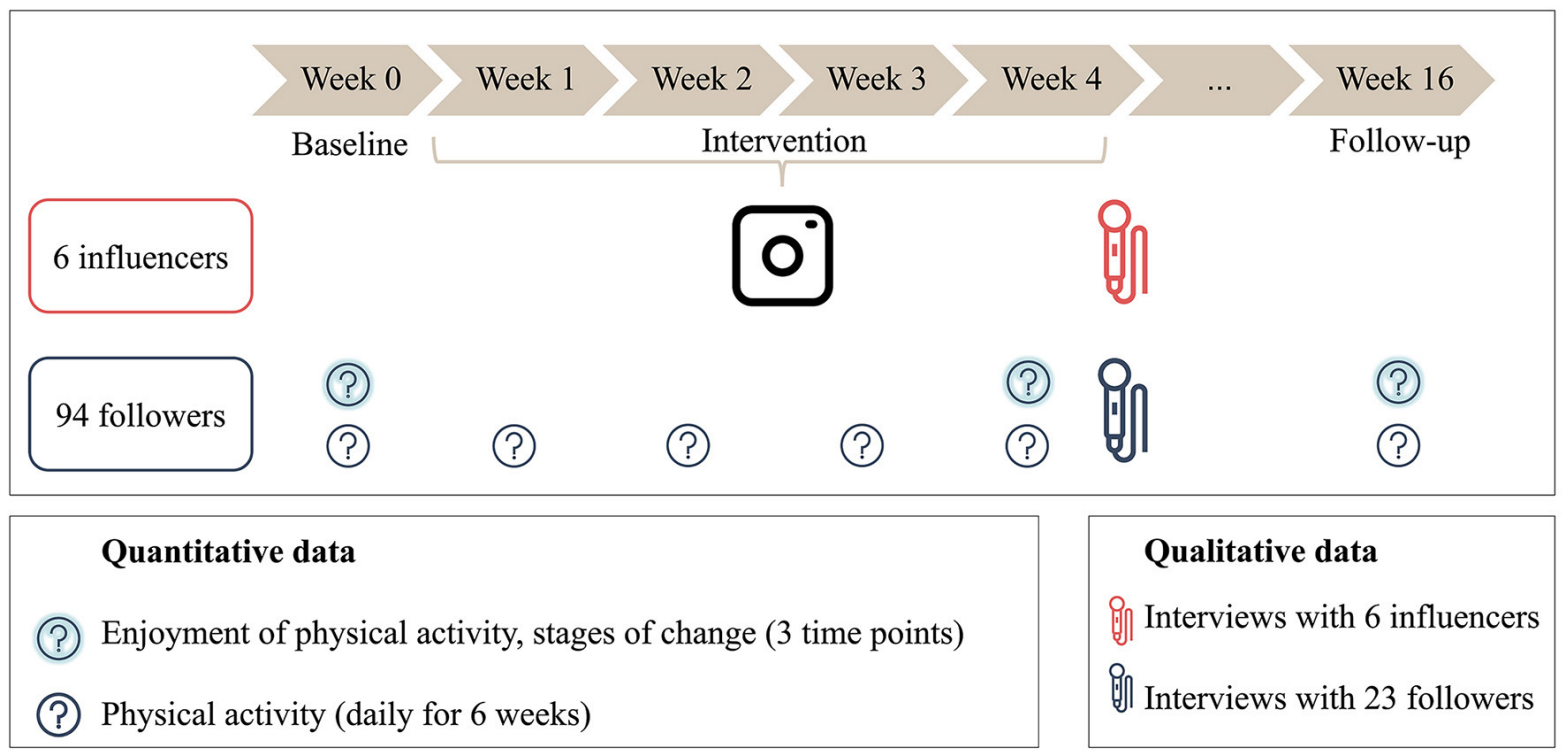
The study procedure consisted of three phases: baseline, intervention, and follow-up. The enjoyment of physical activity and the stages of change were assessed at three different time points, and physical activity was assessed daily. Qualitative data were collected after the intervention (illustration by the authors).

The project was divided into three phases: baseline, intervention, and follow-up (see Figure 2). During the baseline week, the followers received no input from the influencers. During the 4-week intervention phase, the followers were repeatedly encouraged to be physically active through various inputs from the influencers via Instagram. During the follow-up, which took place 3 months after the intervention (week 16), the followers did not receive any more input.

The design incorporated both within-subject and between-subject analyses to comprehensively assess the impact of the social media intervention. For the within-subject aspect, a repeated measures design was used that allowed for the study of enjoyment, stages of change, and physical activity over time within each participant. A between-subject analysis was conducted to explore the differences among the six influencers. This aspect aimed to identify possible differences in participants' enjoyment, behavior change stages, and physical activity associated with specific influencers.

Enjoyment of physical activity and stages of change were measured at three different time points: baseline, after the intervention, and 3 months after the intervention during the follow-up. Physical activity was assessed daily for a total of 6 weeks, including the baseline week, 4 weeks of intervention, and 1 follow-up week (see Figure 2). The adolescents completed the questionnaire online using the link that the project team shared daily on Instagram.

In addition to the quantitative assessments, semi-structured in-depth interviews were also conducted at the end of the intervention. The combination of both within-subject and between-subject designs, along with semi-structured in-depth interviews, enabled a holistic exploration of the intervention's impact on followers.

### 2.3 Materials

In the quantitative data collection, the study assessed three dependent variables: enjoyment of physical activity, stages of behavior change, and physical activity (see Figure 2). Sociodemographic variables, such as age and gender, were assessed at the beginning of the study.

#### 2.3.1 Enjoyment of physical activity

To assess enjoyment of physical activity, we used one item from the German translation of the Physical Activity Enjoyment Scale [PACES, (53)]. The participants rated their agreement with the item “When I am physically active, I enjoy it” on a 5-point Likert scale (1=*disagree at all*, 5=*agree completely*).

#### 2.3.2 Stages of behavioral change

The transtheoretical model outlines five distinct stages in the process of behavior change: The precontemplation, contemplation, preparation, action, and maintenance stage (44). To assess the stages of behavioral change, we used the Physical Activity Stages of Change Questionnaire (54, 55). The questionnaire assesses regular physical activity based on the World Health Organization definition for children and adolescents aged 5–17 years (56). The item asked whether participants engaged in physical activity for at least 60 min per day, seven days a week, with an intensity level sufficient to increase their heart rate slightly and cause mild sweating. The item consists of five response options corresponding to the five behavior change stages. These range from “No, and I do not intend to start regular physical activity in the next 6 months” to “Yes, I have been regularly physically active for more than 6 months” (see Figure 1).

#### 2.3.3 Physical activity

To assess physical activity, duration, and intensity, we used questions based on the Godin–Shephard Leisure Time Physical Activity Questionnaire (57, 58). Participants were asked to report all their physical activities during their leisure time on the assessment day (i.e., “What physical activities did you do today?”). The participants were also asked about the duration in minutes of these activities (i.e., “How long did you do these activities?”). Participants rated the intensity of each physical activity on a 3-point Likert scale (1 = *light*, 2 = *moderate*, and 3 = *vigorous*). Light physical activity was described as nonstrenuous, causing the heart to beat normally and not resulting in sweating. Moderate physical activity was described as a little strenuous, causing the heart to beat slightly faster and resulting in little sweating. Vigorous physical activity was described as strenuous, causing the heart to beat rapidly and resulting in sweating.

#### 2.3.4 Qualitative data collection

We collected qualitative data to gain a deeper understanding of the results of the quantitative data. We conducted semi-structured in-depth interviews in Swiss German via Microsoft Teams with *N* = 23 followers and *N* = 6 influencers at the end of the intervention. The followers could register for the interviews via Instagram. At least three followers from each of the six influencers participated in the interviews. The participants were informed that the interviews were voluntary and that they could stop them at any time. They were informed that the interviews would be recorded with Microsoft Teams. The participants were assured that their data would be anonymized. On average, the interviews lasted 45 min.

The interview guide for both followers and influencers employed semi-structured questions to elicit detailed information. The interview questions for followers are based on self-determination theory, which highlights the importance of autonomy, competence, and relatedness in shaping motivation and behavior (59). These questions aim to gather insights into followers' motivators for and barriers to physical activity and whether these motivators and barriers changed during the intervention; the influencer's characteristics and contents; the project's impact; and the potential influence of social media on adolescents.

The influencers' interview guide covered topics such as their social media activities, strategies, and behaviors; their physical activities; the content they created for the project; how they communicated and interacted with adolescents; the challenges they faced; the potential of the project idea; and how the project impacted them personally (see the Supplementary material interview guides).

### 2.4 Integration of analysis

In this QUAN + QUAL convergent mixed methods study, we initially analyzed the data from the quantitative and qualitative phases separately.

To analyze the effect of the intervention on enjoyment, stages of change, and physical activity, we performed a series of linear mixed effects models. All analyses were conducted using RStudio [version 2022.02.3, (60)], using the R package lme4 [version 1.1-33, (61)].

To analyze the qualitative data, we performed an inductive/deductive hybrid thematic analysis. This hybrid thematic analysis involves a deductive top-down approach, which includes the application of predefined themes based on our interview guides and the transtheoretical model. Additionally, it incorporates an inductive bottom-up approach, which involves generating themes directly from the data itself. This combination has allowed us to draw from an established theory while also capturing themes organically from the data (62, 63).

Each interview was fully transcribed using MAXQDA (64). We predetermined a coding manual with code categories and subcodes based on our interview guides and the literature (deductive approach). The codes were described to indicate which segments corresponded to which code. In the inductive approach, we followed a widely recognized six-stage process, as outlined by Braun and Clarke (65). These stages can be summarized as follows: becoming familiar with the data, creating initial codes, identifying initial themes, reviewing those themes, defining the themes, and conducting the final analysis. Two researchers familiarized themselves with the 29 interview transcripts and first piloted the coding manual on a section of the data. Then, both authors looked independently for data that supported the predefined code categories and subcodes. The coding manual was thoroughly reviewed and adjusted during this process. New codes were created, merged, split, and renamed (inductive approach). Then, both researchers actively constructed initial themes with the analytical research questions in mind. Themes were subsequently modified, adjusted, or merged as we analyzed the remaining transcripts. Illustrative quotes were used to emphasize the four identified key themes. Descriptive memos were simultaneously created on things that appeared important (66). The memos helped us gain a deeper understanding of the data and develop analytical arguments underlying the themes.

After analyzing the quantitative and qualitative data separately, we integrated the results through a narrative process to address key constructs related to the impact of social media influencers on physical activity among adolescents (48). During the integration process, we assessed the data's fit to determine whether there was confirmation, expansion, or discordance between the quantitative and qualitative data (48, 67). Confirmation occurred when both quantitative and qualitative findings aligned and reinforced each other. Expansion was identified when the results provided different insights into the same phenomenon, thereby enriching our understanding. Discordance indicated contradictions between the quantitative and qualitative results. These findings were then presented in joint displays, highlighting how the integrative approach contributed to our meta-inferences based on the combined interpretation of both datasets (68).

## 3 Results

### 3.1 Enjoyment of physical activity (RQ1)

To analyze RQ1_quan_, that is, the enjoyment of physical activity over time, we conducted a linear mixed model. The fixed factor was time, with three factor levels: baseline, after-intervention, and follow-up. We compared the baseline measurements with the after-intervention measurements to assess short-term effects and the baseline measurements with the follow-up measurements to assess long-term effects. We added the covariates gender and age to the model. The model accounted for the nested structure of the data by including a random intercept for the participants.

Over time, followers experienced an increase in enjoyment of physical activity, with greater enjoyment after the intervention (*b* = 0.19, *SE* = 0.08, *t* = 2.30, *p* = 0.002) and at follow-up than at baseline (*b* = 0.23, *SE* = 0.08, *t* = 2.74, *p* = 0.007; see Tables 2, 3).

**Table 2:** Parameter estimates for the mixed effects model.

**RQ1: Effect of time on enjoyment**				
**Fixed effects**	***B***	***95% CI***	***t-value***	***p***
(Intercept)	1.26	−0.24 to 2.77	1.65	0.099
Time [after-intervention]	0.19	0.03 to 0.36	2.30	0.022
Time [follow-up]	0.23	0.06 to 0.39	2.74	0.007
Follower gender [female]	−0.26	−0.57 to 0.06	−1.58	0.114
Age	−0.00	−0.09 to 0.09	−0.06	0.953
**Random effects**				
σ^2^	0.30			
τ_00__participants_	0.40			
ICC	0.57			
N _participants_	92			
Observations	264			
Marginal R^2^/Conditional R^2^	0.033/0.581			

**Table 3:** Mixed methods joint display of quantitative and qualitative findings.

**RQ1_quan+qual_**	**Quantitative findings**	**Qualitative findings**	**Meta-inferences**
Can influencers enhance the enjoyment of physical activity in adolescents?	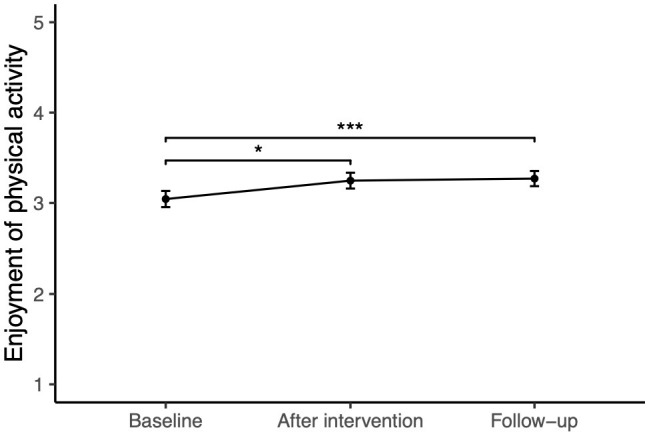 Over time, followers experienced an increase in enjoyment of physical activity, with greater enjoyment after the intervention (*b* = 0.19, *SE* = 0.08, *t* = 2.30, *p* = 0.002) and at follow-up than at baseline (*b* = 0.23, *SE* = 0.08, *t* = 2.74, *p* = 0.007; see Table 2).	**Theme 1: Influencers' genuine enjoyment and enthusiasm are contagious to their followers** Now I find more joy in being active. **(Follower from influencer 4)** It can definitely have an impact if someone posts a video of what they did and shows that it was fun, making others want to try it too. **(Follower from influencer 4)** It really has to look like it's fun for me to want to try it. **(Follower from influencer 2)** You can see that he really enjoys it himself and he's very authentic, you can see his fun behind it, his motivation. **(Follower from influencer 4)** When an influencer genuinely promotes something that they personally enjoy and are interested in, it feels much more impactful. I believe it makes a difference and encourages people to also consider it positively: If they enjoy it and find it worthwhile, maybe I should give it a try too. **(Follower from influencer 3)** If you just go jogging and then say I went jogging for an hour and you are completely tired, it is maybe a little bit different than if you also show a little bit of fun. **(Follower from influencer 4)** Ultimately, sustainable increases in physical activity come from finding something that is genuinely enjoyable and suits the individual. **(Influencer 4)**	**Confirmation**. The quantitative and qualitative results align. Influencers can indeed convey this enjoyment effectively; if they present content authentically and genuinely demonstrate their own enjoyment of physical activity, it can be contagious.

#### 3.1.1 Theme 1: Influencers' genuine enjoyment and enthusiasm are contagious to their followers

To answer RQ1_qual_–whether influencers can affect adolescents' enjoyment of physical activity—we analyzed the interview data. The followers emphasized that the influencers' content must authentically appear enjoyable to impact them. If physical activity is portrayed as strenuous or unappealing, adolescents are unlikely to imitate it. Therefore, the content must look fun to inspire adolescents to try it themselves (see Table 3).

It can definitely have an impact if someone posts a video of what they did and shows that it was fun, making others want to try it too. **(Follower from influencer 4)**

### 3.2 Stages of behavioral change (RQ2)

To answer RQ2_quan_–whether influencers can change the intention to engage in physical activity in adolescents—a linear mixed model was employed to examine the relationship between the response variable stages of change and the fixed effects of time, gender, and age. We added a random intercept for participants to account for repeated measures.

The linear mixed model fit revealed an increase of the stages between the baseline measurement and the after-intervention measurement (*b* = 1.23, *SE* = 0.15, *t* = 8.31, *p* < 0.001) as well as between the baseline and follow-up measurements (*b* = 1.32, *SE* = 0.15, *t* = 9.03, *p* < 0.001; see Table 4).

**Table 4:** Parameter estimates for the mixed effects model.

**RQ2: Effect of time on the stages of change**				
**Fixed effects**	***B***	***95% CI***	***t-value***	***p***
(Intercept)	3.25	1.25 to 5.24	3.21	0.001
Time [after-intervention]	1.23	0.94 to 1.52	8.31	< 0.001
Time [follow-up]	1.32	1.03 to 1.61	9.03	< 0.001
Follower gender [female]	−0.35	−0.77 to 0.07	−1.64	0.102
Age	−0.04	−0.16 to 0.07	−0.74	0.462
**Random effects**				
σ^2^	0.90			
τ_00__participant_	0.54			
ICC	0.37			
N _participant_	91			
Observations	255			
Marginal R^2^/Conditional R^2^	0.218/0.511			

The figure in Table 5 indicates that, at the beginning of the study, 26% of the adolescents reported no intention to start physical activities in the next 6 months (precontemplation stage). By the end of the study, this number had dropped to only 7% and 8%, respectively, in the follow-up evaluations.

**Table 5:** Mixed methods joint display of quantitative and qualitative findings.

**RQ2_quan+qual_**	**Quantitative findings**	**Qualitative findings**	**Meta-inferences**
Can influencers change the intention to engage in physical activity in adolescents?	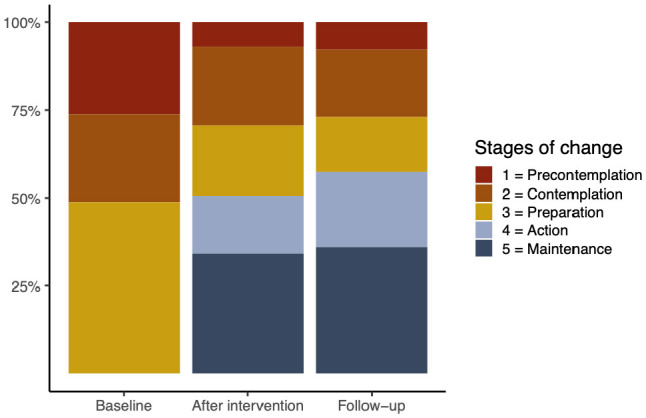 The linear mixed model fit revealed an increase of the stages between the baseline measurement and the after-intervention measurement (*b* = 1.23, *SE* = 0.15, *t* = 8.31, *p* < 0.001) as well as between the baseline and follow-up measurements (*b* = 1.32, *SE* = 0.15, *t* = 9.03, *p* < 0.001; see Table 4).	**Theme 2: Influencers act as a final boost when intrinsic motivation is present** If you have always internally felt the need to be more athletic and then you see people doing sports, you are more likely to switch. **(Follower from influencer 4)** I believe that people who are basically motivated to change something. […] You can only help someone who has already made that decision for themselves. **(Influencer 3)** I already had the motivation to do more sports beforehand, and the project just gave me an extra boost. **(Follower from influencer 5)** [...] you have to have a bit of ambition that you want to do it. In addition, you also have to have the motivation and the desire to do it […]. **(Follower from influencer 4)** [...] I think at the end of the day, these kids or adolescents truly have to figure out something for themselves, a trigger or something that gives them that intention ‘I want to change'. **(Influencer 3)** People who actively want to change something in their life and optimize something. I think the internal motivation has to be there already. I do not think someone who does not truly feel like it is going to change anything. **(Influencer 2)** [...] in the end, you can only help someone who actually wants to move. There are truly these people who just watch something to be entertained, and others who truly want to actively change something in their lives. In addition, I think these are two completely different types. The latter, I do not think you are going to catch that one, the one who actually just consumes. **(Influencer 3)**	**Discordance**. A trend is observed indicating that adolescents' intention to be active has increased, which the quantitative results partially explain. These results suggest that a certain level of baseline motivation is necessary for influencers to have an effect. This implies that individuals in the precontemplation stage may not be influenced by such interventions, as they lack the intention to change.

At baseline, 49% reported being in the preparation stage and were thus willing to start regular physical activity within the next 30 days. After the intervention, the percentage decreased to 20% and 18%, respectively, during the follow-up.

At baseline, none of the participants reported already being in the action or maintenance stage and thus being active for 1 h per day, as this was an exclusion criterion. At the end of the intervention, 51% reported being in regular physical activity (i.e., being in the action or maintenance stage), and at follow-up, 57% did so (see Table 5).

These quantitative results appear promising but must be interpreted with caution. This caution stems from the fact that adolescents were initially in the precontemplation, contemplation, or preparation stages. Consequently, it is not realistic to expect that they would move into the maintenance stage within such a short period of the project, as maintenance typically involves sustained activity over 6 months.

#### 3.2.1 Theme 2: Influencers act as a final boost when intrinsic motivation is present

To answer RQ2_qual_–whether influencers can change the intention to engage in physical activity in adolescents—we also analyzed the interview data. Followers' intention and motivation to change play a crucial role in their receptiveness to influencer content, particularly regarding the transition from online to offline engagement. The interviews revealed that followers who were already motivated and had the intention to change their behavior at baseline were more likely to shift their physical activity from the virtual to the real world and respond positively to influencer content. Both followers and influencers mentioned the necessity of preexisting intention for change (i.e., to be in the contemplation or preparation stage).

If you have always internally felt the need to be more athletic and then you see people doing sports, you are more likely to switch. **(Follower from influencer 2)**

I believe that people who are basically motivated to change something. In addition, that is such an enormous thing. You can only help someone who has already made that decision for themselves. **(Influencer 3)**

I already had the motivation to do more sports beforehand, and the project just gave me an extra boost. **(Follower from influencer 5)**

When adolescents find themselves in the contemplation or preparation stage, influencers can serve as the final boost to initiating action. Interestingly, the quantitative results revealed that some adolescents at baseline lacked the intention to change (precontemplation stage; see Table 5). Nevertheless, during the second and third measurements, there were fewer individuals in the precontemplation stage. This suggests that the intention to engage in physical activity shifted for some, although it cannot be definitively attributed to the influencers because other reasons may have contributed to this change. The reasons for participating in the project varied. Some mentioned that they participated because of their interest in improving fitness, because of their peers, or because of financial incentives. The project resonated most with adolescents who had a genuine interest in physical activity and were waiting for an external trigger to prompt behavioral change. For those who were extrinsically motivated by factors such as financial incentives, the impact on physical activity was limited. These adolescents, engrossed in captivating Instagram videos, often found themselves in prolonged sedentary positions.

Honestly, I'm not sure if I would have participated in the project if there hadn't been any payment for it, I have to admit. **(Follower of influencer 4)**

The more you look online, the more ideas you get about what you could do, but at the same time, it's a bit of a vicious circle because you end up spending a lot of time just looking at these things, and during that time, you're mostly not taking any action. **(Follower of influencer 2)**

These adolescents enjoyed watching sports videos but lacked the motivation to move more or were already satisfied with their physical activity habits. Accordingly, the influencers' content did not have a large impact on them or their physical activity behavior.

### 3.3 Influencers and content that inspire adolescents to increase their physical activity (RQ3)

To address RQ3_quan_–which influencer characteristics and content inspire adolescents to be more physically active—we analyzed the adolescents' physical activity behaviors over time in relation to the influencer they followed. The number of followers per influencer was too small to compare the effectiveness of the six influencers individually (*M*_*number of followers*_ = 16, *SD* = 4.9). Therefore, the quantitative analysis for testing RQ3_quan_ focused on common factors, such as the influencer's athletic background.

#### 3.3.1 Moderating role of influencer athleticism in physical activity

To investigate whether the influencers' athleticism of the influencer moderates the effect of time on physical activity, a regression model was developed. The average daily physical activity time for each participant, regardless of intensity, was calculated. Physical activity was entered as the response variable into the model (repeated measure), with the inclusion of time, influencer athleticism, and their interaction as fixed effects. The participants' gender and age were added as covariates to the model. We specified the random structure of the regression by entering a random intercept for the participants. The model was estimated using the restricted maximum likelihood (REML) criterion. The residuals of the model were normally distributed.

Analysis of deviance (type III Wald F tests with Kenward–Roger *df* ) revealed an effect of time (*F* = 8.52, *p* = 0.004) and an interaction between time and influencer athleticism (*F* = 6.61, *p* = 0.01). The analysis revealed an average increase in physical activity over time (*b* = 4.00, *SE* = 1.37, *t* = 2.92, *p* = 0.004). Interestingly, the interaction revealed that followers of nonathletic influencers demonstrated greater physical activity over time than followers of athletic influencers (*b* = 13.47, *SE* = 2.28, *t* = −2.57, *p* = 0.01; see Tables 6, 7).

**Table 6:** Parameter estimates for the mixed effects model.

**RQ3: Moderating role of the athleticism of influencers**				
**Fixed effects**	***B***	***95% CI***	***t-value***	***p***
(Intercept)	243.07	124.43 to 361.71	4.02	< 0.001
Time	4.00	1.31 to 6.68	2.92	0.004
Influencer athleticism [athletic]	16.26	−12.18 to 44.69	1.12	0.262
Follower gender [female]	−18.49	−43.49 to 6.51	−1.45	0.147
Age	−7.58	−14.60 to −0.56	−2.12	0.034
Time × Influencer athleticism [athletic]	−5.80	−10.24 to −1.37	−2.57	0.010
**Random effects**				
σ^2^	1,917.15			
τ_00__participants_	2,838.28			
ICC	0.60			
N _participants_	93			
Observations	552			
Marginal R^2^/Conditional R^2^	0.058/0.620			

**Table 7:** Mixed methods joint display of quantitative and qualitative findings.

**RQ3_quan+qual_**	**Quantitative findings**	**Qualitative findings**	**Meta-inferences**
Which influencer characteristics and content inspire adolescents to be more physically active?	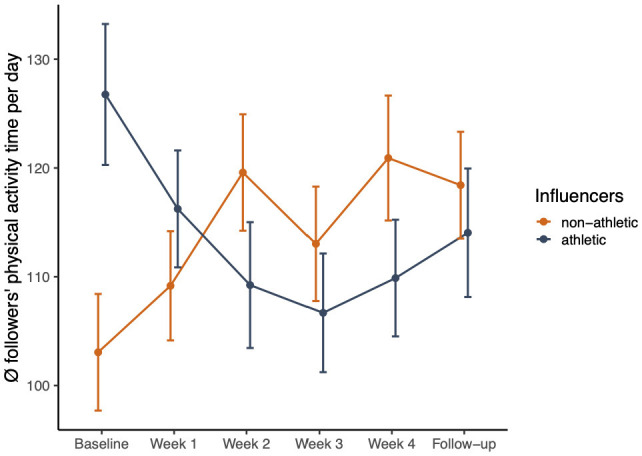 The linear mixed model fit revealed an average increase in physical activity over time (*b* = 4.00, *SE* = 1.37, *t* = 2.92, *p* = 0.004). Interestingly, the interaction revealed that followers of nonathletic influencers demonstrated greater physical activity over time than followers of athletic influencers (*b* = 13.47, *SE* = 2.28, *t* = −2.57, *p* = 0.01; see Table 6).	**Theme 3: Identifying with influencer lifestyle and authenticity serves as an impetus to increase physical activity** We should put the whole society, also normal people, online. However, on social media, that is just even intensified, so this is the ideal image of these athletes. **(Follower from influencer 4)** It should appeal to you because otherwise it is like a movie, and it just runs in front of you. **(Follower from influencer 1)** [Influencer 2] once posted something saying, “Take the stairs instead of the elevator.” In addition, I thought that I still described it well. In my everyday life, I always had it a little bit in the back of my mind. In addition, I do not know if I did more sports blocks like that in general. I rode my bike like two to three times more a week than I might have otherwise. However, those little things take the stairs instead of the escalator. Or that I volunteered to get up and empty the bucket or something. Those things that she brought up in her story. I definitely changed those I think. **(Follower from influencer 2)** It was not big challenges, where I felt like I was running out of breath. In addition, I think [influencer 2] encouraged that, too, the little things. I think when it is the little things, they last longer. **(Follower from influencer 2)** I like to watch them mountain biking, but if I had to go down there myself, I think I would be scared. **(Follower from influencer 6)** That you keep it simple. I think many people do not want mega complicated things, with equipment, and so on. Just that you have a few sympathetic influencers and say, “Go to the swimming pool for a bit.” **(Follower from influencer 5)** He just sits in front of this camera and does it just for the money and because of the product. Then, I feel like it does not work at all. **(Follower from influencer 3)** She interacted with us as if we were at eye level with her. You can also relate. She tries to motivate us and to participate herself. **(Follower from influencer 2)** If you just go jogging and then say I went jogging for an hour and you are completely tired, it is maybe a little bit different than if you also show a little bit of fun. **(Follower from influencer 4)**	**Expansion**. The qualitative findings align and explain why followers of nonathletic influencers increased their physical activity over time and why athletic influencers showed less impact.

#### 3.3.2 Theme 3: Identifying with influencer lifestyle and authenticity serves as an impetus to increase physical activity

Regarding RQ3_qual_, which explores which influencer characteristics and content inspire adolescents to be more physically active, the interview findings showed diverse insights. Influencers who resemble adolescents' everyday lives are more likely to be influential and comprehensible role models. Professional athletes are exceptional talents, and they have the luxury of time to train intensively, which is a luxury that adolescents rarely have because of their school commitments. Thus, it is easier to relate to ordinary people who act as influencers because they lead comparable lifestyles and sometimes display human frailties. For example, if speech mistakes are made in a post, this is seen as authentic and human. In contrast, the artificial portrayal of an idealized image characterized by discipline and a perfect appearance is often far from reality and does not appeal much to adolescents (see Table 7).

We should put the whole society, also normal people, online. However, on social media, that is just even intensified, so this is the ideal image of these athletes. **(Follower from influencer 4)**

Simple and less vigorous activities are well received by adolescent followers. Adolescents are motivated by content that can easily be integrated into their daily lives, such as choosing stairs over the elevator or cycling to school instead of taking the bus. Simple activities require little extra time, which suits the daily lives of adolescents. The greatest obstacle to more physical activity is the stressful everyday life of adolescents, which hardly allows for extra workout.

[Influencer 2] once posted something saying, “Take the stairs instead of the elevator.” In addition, I thought that I still described it well. In my everyday life, I always had it a little bit in the back of my mind. In addition, I do not know if I did more sports blocks like that in general. I rode my bicycle like two to three times more a week than I might have otherwise. However, those little things take the stairs instead of the escalator. Or that I volunteered to get up and empty the bucket or something. Those things that she brought up in her story. I definitely changed those, I think. **(Follower from influencer 2)**

It is fascinating for adolescents to watch impressive videos of challenging activities, such as tricks on trampolines or mountain bikes. They are fascinated by what the influencers are capable of. The problem is that the activities are too demanding for most adolescents to carry out. Adolescents enjoy consuming the content but do not exercise more because of such content. Thus, the adolescents found it easier to engage in everyday activities than to engage in more demanding activities because of time and competences. This is typically not the case with challenging activities, such as tricks on a trampoline or a mountain bike (see Table 7).

I like to watch them mountain biking, but if I had to go down there myself, I think I would be scared. **(Follower from influencer 6)**

Followers mention different content that appeals to them, as well as content that has little impact on them. When content is perceived as authentic, followers are more motivated to move than when content is posed. Remarkably, adolescents can accurately identify whether an influencer's motivation is primarily based on financial gain or a genuine passion for providing meaningful content.

He just sits in front of this camera and does it just for the money and because of the product. Then, I feel like it does not work at all. **(Follower from influencer 3)**

Successful influencers understand the importance of sharing authentic content that fits their persona and stands out from standard content; they try to deliver creative, humorous, entertaining, and personal content to strengthen relationships with followers. Followers appreciate when influencers engage with them by replying to their messages, reposting their content, or receiving positive feedback through personal interactions.

She interacted with us as if we were at eye level with her. You can also relate. She tries to motivate us and to participate herself. **(Follower from influencer 2)**

If influencers share posed content or rarely engage with their followers, these followers believe that the influencer may not genuinely prioritize the promotion of physical activity. As a result, the influencer's content fails to have a meaningful impact or inspire their young audience (see Table 7).

## 4 Discussion

The present study aimed to assess the effectiveness of six carefully chosen social media influencers in motivating inactive adolescents to increase their physical activity. Changing adolescents' physical activity levels is a complex process that occurs across different stages (44). Reaching an inactive target audience is a crucial factor in initiating change. Influencers are well suited to reaching adolescents because they spend much time on social media platforms, using them for both entertainment and information (10). Furthermore, influencers understand their audience and customize their content accordingly, fostering trust and relatability (69). By sharing authentic content within their claimed expertise, influencers create a sense of closeness, often leading to one-way parasocial relationships with their followers (33, 34). This proximity grants influencers motivating power, which is crucial for addressing the lack of motivation among physically inactive adolescents (35). In addition, according to social learning theory, influencers can significantly influence their followers' decision-making and motivate specific behaviors (43). Therefore, it is common and effective for brands to partner with influencers to endorse their products (11–13, 15). Only a few influencers consciously promote the health of their audience (16, 24), for example, their physical activity (25). Although influencers have proven effective in marketing and health promotion, their potential in the context of physical activity among inactive followers remains unexplored. Employing a mixed methods approach, the current study provides preliminary insights into how influencers can contribute to the promotion of physical activity among inactive adolescents.

### 4.1 Influencers increased adolescents' enjoyment of physical activity

The present study's data gave evidence that the six influencers increased the enjoyment of physical activity among adolescents over time. This is significant given that the enjoyment of exercise is an important motivator for engaging in physical activity (35) and is also essential for adolescents to maintain physical activity (70, 71). Furthermore, the enjoyment of physical activity can foster positive affective states (72, 73). In this study, while we did not specifically measure the mediators responsible for the increase in followers' enjoyment of physical activity, the qualitative data suggest that influencers' genuine enjoyment and enthusiasm and the fun they display can positively influence their followers' attitudes toward exercise. This suggests that influencers who authentically express their passion for physical activity can indeed make it more attractive and enjoyable for their followers.

Moreover, the content presented by the influencers often highlighted that physical activity does not need to be limited to intense workouts. By broadening the definition of physical activity to include everyday activities, such as cycling or walking to school, influencers helped shift the perception among adolescents. These activities, which are easier to incorporate into daily life and are often perceived as enjoyable, were shown to be effective in promoting a more positive attitude toward staying active. This approach aligns with the idea that sustainable increases in physical activity are more likely to occur when individuals find activities they genuinely enjoy and can easily fit into their daily routines (74, 75).

### 4.2 Influencers may have changed adolescents' intentions to be physically active

The quantitative data provide evidence that the stages of behavioral change, according to the transtheoretical model (44), have expanded over time in adolescent followers. At the beginning of the study, all participants were in the precontemplation, contemplation, or preparation stage of behavior change. After the intervention, half of the participants categorized themselves as being in the action or maintenance stages. However, this result should be interpreted with caution. The maintenance stage typically requires at least 6 months of consistent activity, which was not achievable within the 4-week intervention period and the 3-month follow-up. Nevertheless, the semi-structured in-depth interviews revealed that the influencers were particularly effective when the adolescents were self-motivated and had the intention to change their physical activity; that is, when they were in the contemplation and preparation stages. The influencers then served as supportive figures, encouraging the desired change in physical activity.

The adolescents who did not make the transition from online to offline and who simply consumed the content were also those who participated for financial reasons. Therefore, motives for participation play an important role and need to be considered in interventions. Adolescents who wanted to improve their fitness or participate with their peers benefited most from the influencers, who acted as external triggers and motivated them to engage in physical activities (76).

### 4.3 Influencers and content that inspired adolescents to increase their physical activity levels

The present study provides interesting quantitative results showing that those followers who followed nonathletic influencers experienced greater increases in physical activity over time, surpassing the levels observed in followers of more athletic influencers. The qualitative findings can provide insights into why the level of influencer athleticism might make a difference. The interviews suggested key elements that positively influenced the behavior of inactive adolescents. Influencers with a similar lifestyle had a stronger positive influence on their followers, which may be due to different underlying reasons. This observation is reflected in social learning theory (77). According to this theory, individuals can acquire skills, behaviors, and attitudes by observing and imitating role models, whereby identification and perceived similarity with these role models are of central importance. In this context, influencers who resemble adolescents' everyday lives are more likely to be influential and comprehensible role models.

This is in line with the main success factor of classic influencer marketing: Influencers who are perceived as congruent with their followers have a more positive attitude toward the influencers, resulting in greater purchase intentions (78). The same principle may also apply to physical activity. It is easier for adolescents to imitate behavior if they can identify with their idols because they have a similar lifestyle. Fewer sports-enthusiastic influencers had to motivate themselves to be more active. They had to think about how they could bring more physical activity into their everyday lives. In other words, they were at the same point as many of their followers. This could also explain why simple everyday activities were better received by adolescents than more demanding activities. Athletic influencers are exceptional talents with a professional sports lifestyle that does not reflect the reality of most followers. They have shown challenging and captivating content, such as flips on a trampoline or tricks on a mountain bike. Inactive adolescents may be motivated to watch entertaining videos, but the content does not affect their intentions to exercise more. Social cognitive theory suggests that this phenomenon might be attributed to social comparison and the perceived inability to perform the behavior being modeled (37). In contrast, the nonathletic influencers focused on simple, everyday physical activities, such as taking the stairs, walking, or cycling to school. As a result, adolescents felt competent in their actions, fulfilling one of the three basic psychological needs outlined in self-determination theory (59). The advantages for adolescents in adopting these simple daily movements were also their nonperspiring nature and minimal time commitment. This is crucial because time is the main barrier to not practicing more regularly (35), especially for adolescents with demanding daily lives. Another reason why some of the followers did not exercise more over time may be related to vicarious goal fulfillment, a phenomenon observed in food consumption. Vicarious goal fulfillment describes how simply looking at healthy food options can paradoxically trigger unhealthy eating behavior (79). Having healthy food available vicariously fulfills nutritional goals, leading consumers to be more likely to choose indulgent options. The same may have happened with the adolescents' physical activities. Watching the videos gave them the vicarious feeling that they had already done some activities and then stayed seated.

Regarding social media content, adolescents considered key characteristics that have influenced them. When influencers' content is perceived as authentic, meaning that it aligns with their persona and other content, they are more likely to motivate behavioral changes. This perceived congruence has also been observed in marketing, highlighting the importance of marketers selecting influencers whose image aligns with their brand (80, 81). Congruence is more important than the influencer's follower count or popularity. Paid content must match the influencer's usual style (82). Interestingly, in our study, the influencers with a sporting background were less successful at motivating adolescents to exercise than the less athletic influencers were. It might not be expected that a musician or a journalist would promote physical activity as they have no expertise in this area. However, they were able to engage adolescents through simple, everyday activities that fit their lifestyles. The adolescents said they were good at distinguishing between authentic and artificial videos, particularly noting instances where the primary motive behind the posts appeared to be monetary gain. An indication of artificial content was when the influencers did not interact at all (i.e., when it was a purely parasocial relationship). Influencers who took the time to turn the parasocial relationship into a reciprocal one were described as particularly effective (81). They interacted with their followers, for example, in group chats or by responding to their reactions. As a result, adolescents listened to and had a sense of belonging, which aligns with the relatedness factor of self-determination theory (59).

Other key characteristics of influential content include being personal (69), being entertaining (10), and creatively different from mainstream content. Otherwise, they may simply continue to swipe because of the overwhelming volume of content. These findings are in line with those of Casaló et al. (83), who have emphasized that originality and uniqueness are essential factors for a user to be perceived as an opinion leader on Instagram. Originality and uniqueness increase consumers' intention to interact with the influencer, follow their advice, and recommend the influencer to others.

### 4.4 Limitations and future directions of the research

Influencers operate in many ways, varying in their topics, presentation styles, relationships with followers, level of professionalism, and posting frequency. In this study, we analyzed six influencers, each with a different impact on their followers' physical activities. Identifying the key factors behind these influences remains an open question. Interestingly, the three nonathletic influencers had a greater effect on follower activity than the athletic ones. Other factors, such as authenticity or the nature of the influencer–follower relationship, might also play a role in these results. While our mixed methods data revealed associations, we could not establish causation due to the study's design—it was not a controlled laboratory experiment or a randomized trial. Conducting experiments that manipulate how influencers operate, without introducing confounding variables, would be challenging but crucial for identifying the exact factors responsible for the observed differences in physical activity.

Our sample includes a wide age range from 14 to 20 years, a period during which there are developmental differences among adolescents. Additionally, there were relatively few adolescent followers per influencer who participated. This is likely due to our stringent criteria, which included age and < 1 h of daily physical activity. Reaching and motivating this inactive target group for a physical activity project is challenging (27), even with financial incentives.

In the future, physical activity data could benefit from incorporating objective measurement methods alongside subjective information. Self-reported measures can overestimate physical activity levels by up to 70% (74). To achieve a more accurate assessment of physical activity, device-based measurements, such as accelerometers or pedometers (for walking activities), are recommended. Objective measurements would also help to better understand the long-term effects of the intervention on behavior change.

In future research, it would be valuable to explore the role of peer influence, particularly in the context of social media. The amplification effect of social media on peer interactions suggests that peers could significantly influence adolescents' behaviors and attitudes by making these interactions more frequent, widespread, and impactful (84). Examining how peer influence can be leveraged to spread positive health promotion messages and create supportive online environments would be a promising direction for further study.

## 5 Conclusion

The present study provided evidence that six influencers were able to enhance the enjoyment of physical activity among inactive followers. Furthermore, key factors on Instagram were identified that encourage inactive adolescents to engage in more physical activity. These factors include the followers' ability to identify with influencers because of similar lifestyles (80), promotion of simple daily physical activities, authenticity of content (82), and followers' intention to change.

Nonathletic influencers can increase physical activity, which is surprising because they usually promote other topics. Even though a musician, a journalist, and an eFootballer shared little physical activity content before the project, their followers perceived their content as authentic. This is because they shared the same goal as their followers, which was to make their everyday lives more active. Accordingly, they promoted simple everyday activities that were well received by adolescents because they were less strenuous, easier to do, and took less time.

This means that future physical activity promotion projects should not rely solely on professional athletes as influencers. Professional athletes often promote activities that are too difficult for followers to imitate (37), which may fail to effectively increase their followers' intentions to engage in more physical activity. Instead, such projects should focus on nonathletic influencers who are motivated to incorporate more physical activity into their daily lives. People tend to follow people with similar lifestyles (26). As a result, less active influencers may have untapped potential because their followers may also be less active. This similar initial situation of influencers and followers provides an opportunity to increase physical activity, especially through everyday activities.

## Data Availability

The datasets presented in this study can be found in online repositories. The names of the repository/repositories and accession number(s) can be found in the article/Supplementary material.
