# Supplementary material for: How influencers motivate inactive adolescents to be more physically active: a mixed methods study
Source: Front Public Health. 2024 Sep 17;12:1429850. doi: 10.3389/fpubh.2024.1429850 (PMC11445662; doi:10.3389/fpubh.2024.1429850)
Supplement: Supplementary file 1 [file Data_Sheet_1.docx]

Supplementary Material

**Interview guide for followers**

**Questions**

- RQ1_qual_: Can influencers enhance the enjoyment of physical activity in adolescents?
- RQ2_qual_: Can influencers change the intention to engage in physical activity in adolescents?
- RQ3_qual_: Which influencer characteristics and content inspire adolescents to be more physically active?

| **0. Administration (0-5’)** |
| --- |

***Goal: greeting, goal of the conversation and procedure***

Good day [x]

Thank you for your willingness to participate in this interview.

*About me: I am ....... and I am a [x] at the Department of [x] of the Institute of [x] at the University of [x].*

Together with [x], we are carrying out this project on the topic of physical activity in the everyday lives of adolescents. This project is part of the [x]. We are interested in whether influencers can motivate their communities to lead an active and healthy lifestyle.

Today [x] and [x] from the [x] are listening so that they get a general impression of the conversation. I am happy to give the floor to [x] to briefly introduce himself/herself.

Person [x]: ...

Thank you very much for introducing yourself. [X] will now turn off his/her sound and camera. She/he may ask further questions at the end.

It is important for us that you know that no information is stored under your name. Your answers can therefore not be associated with your name.

The idea is that you share your opinion and experiences with us as honestly as possible. There is no right or wrong, or particularly clever or less clever answers.

The interview is voluntary, and you can stop it at any time. If a question is not understandable or you do not want to answer it, you can say so at any time.

The planned duration of the interview is a maximum of **45 minutes**. You will be rewarded with an additional CHF 50.-. This means that after the follow-up measurement in autumn, you or your parents will receive the total amount of CHF 145.-.

We will record the conversations so that we can evaluate them afterwards. Is that okay with you? Then I will start the recording now.

**[start recording]**

| **I. Entry (5-10’)** |
| --- |

***Goal****: General acquaintance, understanding of the current situation of the interviewee.*

**Everyday life of the interviewee**

- First, can you tell me something about your current daily life?
  - - *Prompt: Education, daily tasks, typical day, family, hobbies*
    - *Prompt: Person and living situation*
- If you had to describe yourself with three hashtags, what would they be?

**Brainstorming**

Then I would like to do a short brainstorming now.

- What is the first thing that comes to mind when you hear the term physical activity?
  - - *Prompt: Image in front of eyes, thoughts, feelings, experiences*

| **II. Sportfluencer project (10-30’)** |
| --- |

**Social Media**

*The following block of questions refers to your general social media activities, i.e., not specifically related to the Sportfluencer project.*

- Do you have preferences in social media channels? Why?
  - *Prompt: TikTok, Instagram, Snapchat, Facebook, WhatsApp*
- What do you do when you are on social media?
  - *Prompt: Mainly input or also consumption?*
  - *Prompt: Be sprinkled, stories, look at subscribed/suggested posts, write content yourself, read/write comments, chat function, out of boredom/interest*
- Do you have any role models on social media? Which ones and why?
- What content do you find particularly exciting?
  - *Prompt: Fitness, sports, nutrition, lifestyle, travel, music, gaming*
- Are there people and content that do not interest you at all? Which ones and why?
- How active are you on social media? Why?

**Motivation**

*The following questions relate to the Sportfluencer project.*

- What was your motivation to participate in the Sportfluencer project?
  - *Prompt: Money, friends, interest, influencer*
- Who brought the project to your attention?

*From the survey we know that you follow influencer [x].*

- Did you follow this person before the project or were you assigned?
- **If already followed:** Can you remember why you wanted to follow this person?
- **If assigned:** Did you know the person before?
- To what extent can you identify with this personality?
  - *Prompt: Identification, lifestyle, videos, role model, admiration*

**Competence and contents**

- What kind of content did you see from the influencer?
  - *Prompt: Strategy, goals, showcase videos, motivational sayings, challenges, own experiences, tips, focus topics*
- How did you come across the influencer's content?
  - *Prompt: Time, explicitly checked Sportfluencer profile, only clicked through stories, randomly*
- Was there any content of the influencer that motivated you to do additional physical activities? Which ones and why (not)?
  - *Prompt: Did you feel competent while watching the content?*
  - *Prompt: Did you feel competent while doing physical activities?*
- *[If yes]* Was there content that only worked for a short time and other content that motivated you longer? Which ones and why (not)?
  - *Prompt: What has been the short-term impact and what has been the longer-term impact?*
- Was there content that appealed to you less or not at all? Which ones and why?
  - *Prompt: Not motivating, disturbing, annoying, irritating, not authentic, other sports*
- Was influencer [x]'s content authentic to you? Why?
- What else would you have wanted from the influencer?
- How did you go about filling out the surveys on Insta?
  - *Prompt: Reminder, before going to sleep, only on Sunday, often forgotten*

**Relatedness with influencer and its characteristics**

- To what extent did you feel that you were influenced?
  - *Prompt: Lifestyle, attitude, team membership, social relationship, physical activity, motivation*
  - *Prompt: Could you relate with the influencer?*
- How did the influencer communicate with you?
  - *Prompt: Close friends, group chat, stories, posts, live, reels, Sportfluencer_Projekt*
- Which forms of communication appealed to you and tended to motivate you? Which ones less?
  - *Prompt: Why did the group chat appeal less to you?*
- How was the exchange/interaction between the influencer and you during this month?
  - *Prompt: Reserved, motivating, authentic/artificial, regular, one-sided/parasocial*
- How was the exchange/interaction with the other participants of the project?
  - *Prompt: Other followers, project team, people from outside the project*

**Autonomy and change of perspective**

- What do you think: Can influencers influence the lifestyle of, for example, your friends (peers)?
  - If so, do the followers still control their behavior of their own free will?
- What do you think: Can influencers motivate your friends to exercise more, for example?
  - *Prompt: About what activities?*
- Which target groups are particularly addressed by influencers?
  - *Age, gender, education, place of residence, country, athletic, non-athletic*
- What do you think: What does it take to get online content not only viewed but acted upon?
  - *Prompt: Which followers make that switch?*
  - *Prompt: With which strategies?*
- What do you think it would take to motivate adolescents who do not exercise much to be more physically active?
  - *Prompt: Online/offline, tutorials, courses, more free time, less phone time*

**Multiplicator**

- Can you imagine putting your physical activities online as well?
  - If so, with what intention?
  - If no, why not?
    - *Prompt: Self-confidence, private, sport level, personality, competence*
- Do you see yourself in the future in the role of positively influencing others with such content? Why (not)?

| **III. Physical activity changes (30-40’)** |
| --- |

**Physical activity afterwards**

«The following questions refer to the time **after** the Sportfluencer project, i.e., from July 2022 onwards.»

*Introduce sentences: Compared to before the project …*

- Has your standard week changed compared to before the project?
  - *Prompt: physical activity, free time, phone time*
- …have your physical activities changed?
  - *Prompt: Changes club, school sports, leisure sports*
  - *Prompt: Fixed dates or spontaneous*
  - *Prompt: Discovered a new sport? Learned new physical activities? Got information? Frequency? Intensity? Habit? Place? "Strategy"?*
- …what motivates you to be physically active today? Have you added any new motivators?
  - *Prompt: Coach, peers, fun, balance, weight loss, muscle gain, social media content, life school, health, achievements*
- …what prevents you from being even more physically active today?
  - *Prompt: Priorities, time, desire, no offer, no friends, no enjoyment, unwell, weather*
- …do you set personal goals that you want to achieve in terms of your physical activities? What are they?
- …how do you feel today during a physical activity?
  - *Prompt: Exhausting, uncomfortable, competent, fear of competition/comparison, shame vs. enjoyment, satisfied, pride*
- …do you feel the desire to be more physically active today?
  - *Prompt: If so, why?*

| **IV. Social media behavior and project info [if enough time]** |
| --- |

- Did you spend more time than usual on your phone during this month?
  - *Prompt: If so, why?*
- Are you yourself more active on social media than you were before the project? Why? *[rather unimportant]*
- Do you want to continue actively following the influencer in the future and implement his/her tips? Resp. does he/she still post in this direction?
- Was the project understandable for you?
  - *Prompt: Procedure, survey, payout, goal, content*
- Has the project been educational for you?
  - *Prompt: If so, in which areas?*
- How is your motivation to participate in a physical activity project again? *[scale from 1-10]*. Explain.

| **V. Conclusion (40-45’)** |
| --- |

***Goal****: Capture the most important points*

- We heard a lot of exciting input from you and many things were addressed. Is there anything that comes to mind around the topic of social media and physical activity that we have not yet discussed?
- Finally, can you give me an «advice»: In your opinion, what should one pay attention to when it comes to «physical activity among adolescents»?
- I am happy to turn the floor over to [x], do you have any questions?
- Do you have any unanswered questions or comments?

Thank you very much for taking the time for this interview. You will hear from us again in October. Then you will be asked again to fill out the survey during one follow-up week from October 3-9, 2022. After that you or your parents will receive CHF 145.-.
