# Supplementary material for: How influencers motivate inactive adolescents to be more physically active: a mixed methods study
Source: Front Public Health. 2024 Sep 17;12:1429850. doi: 10.3389/fpubh.2024.1429850 (PMC11445662; doi:10.3389/fpubh.2024.1429850)
Supplement: Supplementary file 2 [file Data_Sheet_2.docx]

Supplementary Material

**Interview guide for influencers**

**Questions**

- RQ1_qual_: Can influencers enhance the enjoyment of physical activity in adolescents?
- RQ2_qual_: Can influencers change the intention to engage in physical activity in adolescents?
- RQ3_qual_: Which influencer characteristics and content inspire adolescents to be more physically active?

| **0. Administration (0-5’)** |
| --- |

***Goal: greeting, goal of the conversation and procedure***

Good day

Thank you for your willingness to participate in this interview.

*About me: I am ....... and I am a [x] at the Department of [x] of the Institute of [x] at the University of [x].*

As you already know, we are running this project together with [x] on the topic of physical activity in the everyday lives of adolescents. We are interested in whether you as influencers can motivate your communities to lead an active and healthy lifestyle.

Today [x] is listening so that he/she can get a general impression of the conversation. I am happy to give the floor to [x] to introduce himself/herself briefly.

Person [x]: ...

Thank you very much for introducing. [X] will turn off her/his sound and camera. She/he may ask further questions at the end.

It is important for us that you know that no information is stored under your name. Your answers can therefore not be associated with your name.

The idea is that you share your opinion and experiences with us as honestly as possible. There is no right or wrong, or particularly clever or less clever answers.

The interview is voluntary, and you can stop it at any time. If a question is not understandable or you do not want to answer it, you can say so at any time.

The planned duration of the interview is a maximum of **45 minutes**.

We will record the conversations so that we can evaluate them afterwards. Is that okay with you? Then I will start the recording now.

**[start recording]**

| **I. Entry (5-10’)** |
| --- |

- What does a standard week look like for you (if there is one)?
  - *Prompt: Education, physical activity, leisure*

**Physical activity**

- What are the main physical activities you engage in?
  - *Prompt: In everyday life, in a club, leisure sports, profession, alone vs. together*
  - *Prompt: Regular fixed dates or spontaneous*
- What motivates you to be physically active?
  - *Prompt: Coach, peers, fun, balance, weight loss, muscle gain, social media content, life school, health, achievements*
- What is hindering you from being more physically active?
  - *Prompt: Time, desire, no offers, no friends, no enjoyment, uncomfortable, weather, money, effort*

**Social Media**

*The following question block is about your general social media activities, i.e., not specific to the Sportfluencer project.*

- Do you have preferences in social media channels? Why?
  - *Prompt: TikTok, Instagram, Snapchat, Facebook, WhatsApp*
- What do you do when you are on social media?
  - *Prompt: Mainly input or also consumption?*
  - *Prompt: Be sprinkled, stories, look at subscribed/suggested posts, write content yourself, read/write comments, chat function, out of boredom/interest*
- How active are you on social media? Why? [rather unimportant]
  - *Prompt: Quantity of content per week*
- How did you come to have such a large following? Was there a beginning?
  - *Prompt: Was it always your goal, was it planned?*
- What is important to you about the content you put online?
  - *Prompt: Authentic, funny, entertaining, interactions, sponsors, money*
- What feelings are aroused in you when you look at your profile with your content?
  - *Prompt: Uncomfortable, funny, insecure, satisfied, proud, indifferent*
- What is your strategy for creating your content?
  - *Prompt: Planning (I go out and know I am producing content), spontaneous (cool, I need to film this), accumulation of videos, editing/music, outside help, timing of posts (automated)?*
- How much time do you spend on a post on average?
  - *Prompt: Difference between standard post vs. sportfluencer post*

| **II. Sportfluencer project (10-30’)** |
| --- |

*The following questions relate to the Sportfluencer project.*

- How did you prepare for the project?
  - *Prompt: To the Roundtable*
  - *Prompt: To the month in which you were active with the project participants?*
- Why did you decide to participate in the Sportfluencer project?
  - *Prompt: Network, money, interest in the project, promote adolescents*

*For the project you were followed by about 20 adolescents.*

- Did you know some of them or were they all strangers to you?
- To what extent did you manage to identify with the idea of the project?
  - *Prompt: 7x1, promote physical activity, motivate adolescents*

**Contents**

- How have you tried to motivate your community?
  - *Prompt: Strategy, goals, showcase videos, motivational sayings, challenges, own experiences, tips, focus topics*
- How did you come up with these ideas?
  - *Prompt: Roundtable, exchange with other influencers / environment, internet*
- How did you approach your content?
  - *Prompt: Timing, regularity/frequency, planned through month, all content planned and produced at once vs. spontaneous ideas, reminders*
- Did your own content also motivate you to move more? Which ones and why (not)?
- Which of your content do you think resonated well with your community? Why?
- Do you think there was content that only had a short impact and content that motivated adolescents for longer? Which ones and why?
- Which of your content do you think resonated less well or not at all with your community? Why?
  - *Prompt: Not motivating, annoying, irritating, not authentic, other sports*

*If you compare your other content with the content for the Sportfluencer project.*

- On what points do they differ?
  - *Prompt: Did you feel authentic?*

**Influence and relationship with community**

- To what extent did you feel like you impacted your community?
  - *Prompt: Lifestyle, attitude, team membership, social relationship, physical activity, motivation*
- How did you communicate/interact with followers?
  - *Prompt: Close friends, group chat, stories, posts, live, reels*
- How was the exchange between you and your community during this month?
  - *Prompt: Reserved, motivating, authentic/artificial, regular, one-sided/parasocial*
- What kind of communication do you think appealed to the adolescents and was more likely to motivate them? Which less?
- How did you perceive your community?
  - *Prompt: Interactions, openness, interest, motivation, questions*

**Motivation**

- What was your motivation to carry out the project? *[Scale from 1-10]*
  - *Prompt: Preparations, roundtable, conversations, emails*
  - *Prompt: During the four weeks*

**Challenge**

- What was challenging for you during the project?
  - *Prompt: Preparations before the month, interaction with adolescents, ideas for content, project understanding*

**Influencer potential in general**

- What do you think: Can influencers influence the lifestyle of their followers?
- Which target groups are particularly addressed by influencers?
  - *Age, gender, education, place of residence, country, athletic, non-athletic*
- What do you think: Can influencer activities lead to more physical activities?
  - *Prompt: If so, with whom?*
  - *Prompt: About what activities?*
- What do you think: What does it take to get online content not only viewed but acted upon?
  - *Prompt: Which followers make that switch?*
  - *Prompt: With which strategies?*
- What do you think it would take to motivate adolescents who does not exercise much to be more physically active?
  - *Prompt: Online/offline, tutorials, courses, more free time, less cell phone time*
- Do you think followers can become addicted to influencers?
  - *Prompt: What level of dependence is still healthy?*
- How could your followers become multipliers, meaning that they positively influence their followers as well? *[rather unimportant]*

| **III. After the project (30-35’)** |
| --- |

**Changes**

*The following questions are about before and after comparison. With the surveys, we asked about physical activity, intention, enjoyment, and overall health perceptions. How do you assess changes in adolescents on these items?*

**Do you think anything has changed since the project started in terms of ...?** *[we can see the quantitative results in the surveys. It might be difficult for them to answer, but maybe they have a feeling for it]*

- ...the follower’s physical activity?
  - *Prompt: Discovered a new sport? Learned new physical activities? Got information? Frequency? Intensity? Habit? Place? Strategy?*
- ...their intention to move more?
- ...their enjoyment of physical activity? Why?
  - *Prompt: Social contacts, ideas, coach*
- ...their overall health perception?
  - *Prompt:* *Physical, mental, and social well-being (not only the absence of illness)*
- How did you perceive your community at the beginning of the project and at the end of the project?
  - *Prompt: Interactions, openness, interest, motivation, questions*

| **IV. Project info (35-40’)** |
| --- |

- When you look back on this month. Have you imagined the project the way it came out? What was unexpected?
- Do you want to continue to be active and provide physical activity tips to the community in the future? Are you still doing so? Why (no longer)?
- What is your motivation to participate in a physical activity project again? *[Scale from 1-10]*. Explain.
- How much would you charge for a similar campaign? [*for this project they get CHF 6'000.-]*
- Was the project understandable to you?
  - *Prompt: Procedure, surveys, payout, goal, content*
- Has the project been educational to you? Learnings?
  - *Prompt: If yes, in what areas?*

| **V. Conclusion (40-45’)** |
| --- |

***Goal****: Capture the most important points*

- We heard a lot of exciting input from you and many things were addressed. Do you have any thoughts about social media and physical activity that we have not yet discussed?
- Finally, can you give me an "advice"? From your point of view, what should one pay attention to when it comes to "physical activity among adolescents"?
- I would like to give the floor to [x], do you have another exciting question?
- Do you have any open questions or comments?

Thank you very much for taking the time for this interview. You will hear from us again in October. Then your followers will have another week to fill out the survey (October 3-9). However, you do not have to be active until then and during that week. After the follow-up week, we will send you a short survey. There you will be asked if and how the project has developed.
